# Air Embolism: Practical Tips for Prevention and Treatment

**DOI:** 10.3390/jcm5110093

**Published:** 2016-10-31

**Authors:** Colin J. McCarthy, Sasan Behravesh, Sailendra G. Naidu, Rahmi Oklu

**Affiliations:** 1 Massachusetts General Hospital, Harvard Medical School, Division of Interventional Radiology, 55 Fruit Street, GRB-290A, Boston, MA 02114, USA; colin.mccarthy@mgh.harvard.edu; 2 Mayo Clinic Arizona, Division of Vascular & Interventional Radiology, Phoenix, AZ 85054, USA; Behravesh.sasan@mayo.edu (S.B.); Naidu.Sailen@mayo.edu (S.G.N.)

**Keywords:** Air embolism, endovascular, catheter, embolization

## Abstract

Air embolism is a rarely encountered but much dreaded complication of surgical procedures that can cause serious harm, including death. Cases that involve the use of endovascular techniques have a higher risk of air embolism; therefore, a heightened awareness of this complication is warranted. In particular, central venous catheters and arterial catheters that are often placed and removed in most hospitals by a variety of medical practitioners are at especially high risk for air embolism. With appropriate precautions and techniques it can be preventable. This article reviews the causes of air embolism, clinical management and prevention techniques.

## 1. Introduction

An intravascular air embolism (VAE) is a rare, preventable, but serious complication of endovascular procedures resulting in significant morbidity and mortality. It occurs as a result of a pressure gradient that allows air to enter the blood stream, which can subsequently occlude blood flow. This complication can arise in a range of clinical situations including interventional radiology (IR) procedures, trauma, barotrauma, central line placement and removal, and certain types of surgical interventions such as cardiac and neurosurgery. The main causes of systemic and cerebral air embolism are shifting from the traditional culprits of open surgery and trauma to endoscopy, angiography, tissue biopsy, and peripheral venous access [1,2]. While the true incidence of air embolism is unknown, as many instances go unreported, one study included a series of over 11,000 central venous catheter placements and found an incidence of 1 in 772 [3]. As a result of its high chance of mortality and morbidity, physicians should be well equipped to prevent, identify, and manage air embolism. In this article, we explore the clinical presentation of air embolism, review practical tips for prevention and treatment, and present cases of iatrogenic air embolism.

## 2. Etiology of Air Embolism

Air emboli exist only when there is a connection between air and the vascular system. A pressure gradient is required to drive air into the vascular system; a central line or its tract (post-removal) is an example of such a connection. Often these lines terminate in the superior vena cava where low central venous pressure (CVP) below atmospheric pressure further increases the likelihood of an air embolism. 

A venous air embolism occurs when air enters the venous system and eventually causes an obstruction in the pulmonary circulation. The gradient between external atmospheric pressure and the intravascular low central venous pressure (CVP) is especially increased by hypovolemia or during inspiration by creating a negative intrathoracic pressure which enhances the possibility of air entry. As CVP may be sub-atmospheric at baseline in up to 40% of patients [4], those patients in an upright position or those undergoing IR procedures such as hemodialysis catheter placements are particularly susceptible.

In contrast, arterial air embolism occurs following direct instillation of air into the arterial tree (i.e., angiography) or paradoxically, through a septal defect or patent foramen ovale (PFO). Though the higher intravascular pressure in the arterial system is somewhat protective, arterial air embolism has the potential to produce ischemia or infarction in any organ with limited collateral blood supply, even when the volume of air is small. 

The consequences of intravascular air entrainment are determined by volume, rate, and route (venous or arterial). Animal studies have been performed to estimate the volume of air required to produce lethal circulatory arrest, with case reports suggesting the lethal dose of air in adults to be between 200 and 300 cc, or 3–5 mL/kg [1,5,6]. Though this appears to be a large amount of air not easily introduced to the vascular system, it has been shown that a 14-gauge needle can transmit 100 cc of air per second with a pressure gradient of 5 cm H_2_0. In addition, a 15 French (5 mm diameter) peel-away sheath used during placement of a tunneled hemodialysis catheter can allow 300 cc of air to enter the vascular system in just 0.5 s [4]. When a large bolus of air lodges in the right ventricular outflow tract (RVOT), this can result in pulmonary hypertension followed by circulatory collapse.

**Figure 1:**
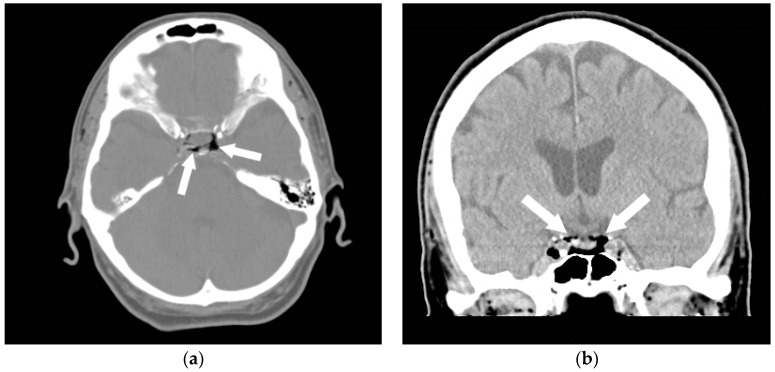

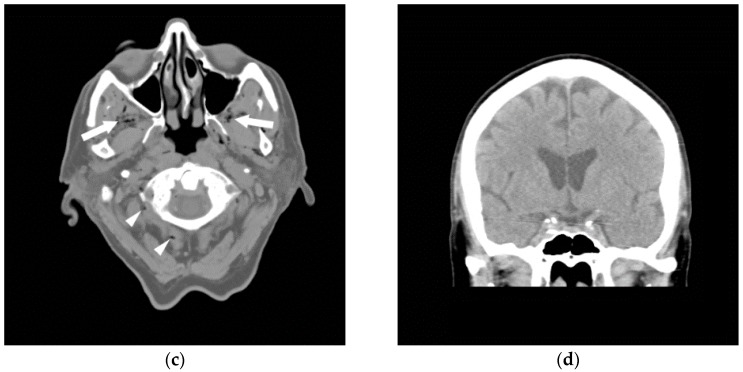
A 35-year-old man was transferred from an outside hospital with altered mental status following the placement of a peripheral intravenous line. A CT of the brain was performed in the emergency room. Axial (**a**) and coronal (**b**) CT revealed a moderate amount of air in the cavernous sinuses bilaterally (arrows) as well as multiple small foci of air in the region of the pterygoid venous plexuses (**c**), scattered in the soft tissues and in the extradural space at the level of the upper cervical spine (arrowheads). A follow-up CT (**d**) was performed the next day, which revealed resolution of the previously seen air. In this case, the patient made a full recovery.

**Figure 2:**
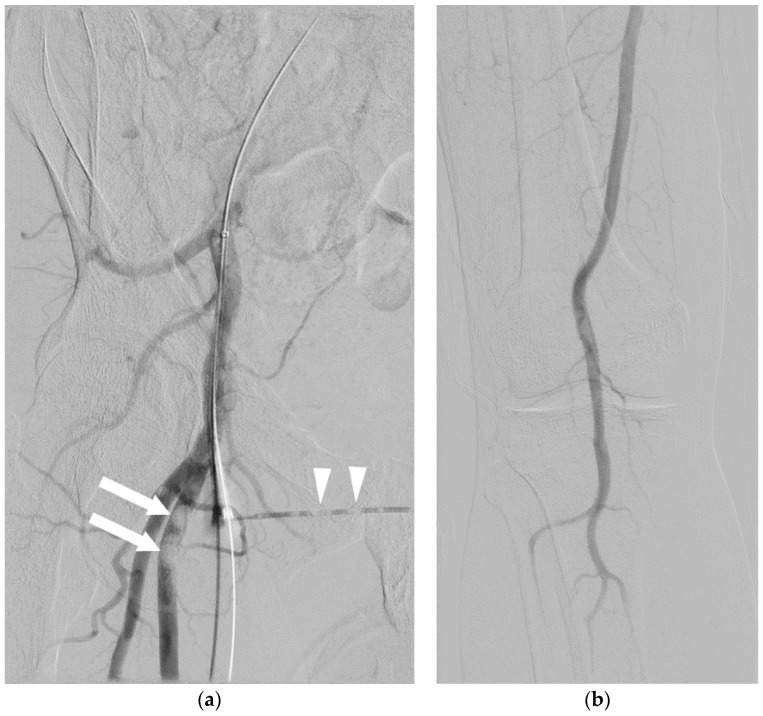
A 61-year-old male underwent an initial femoral angiogram (**a**), which revealed a small amount of air in the tubing of the side port (arrowhead) that had traveled into the superficial femoral artery (arrows). As the lower extremity arteriogram (**b**) revealed no visible distal emboli, it was presumed the air was resorbed. There were no adverse sequelae.

**Figure 3:**
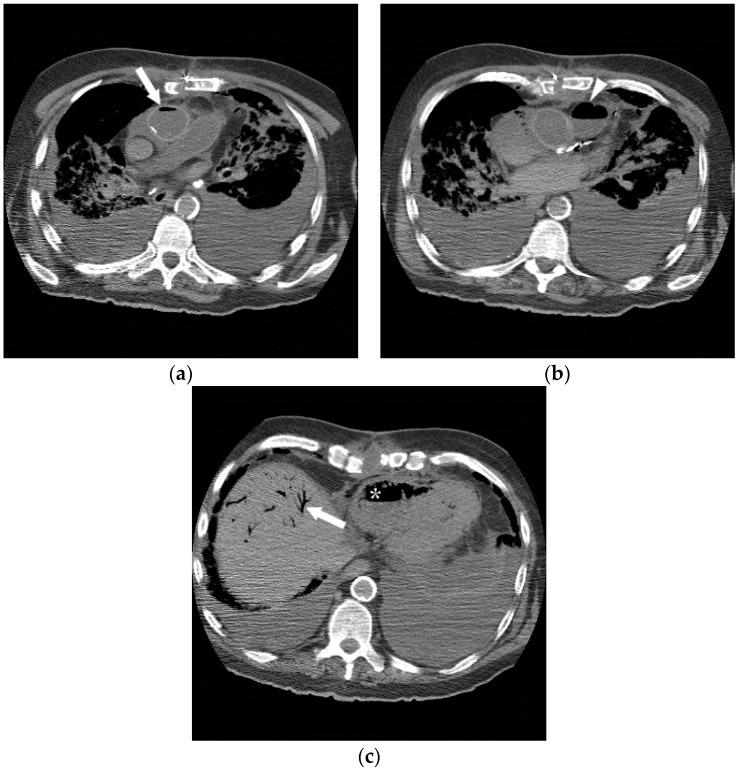
A 69-year-old man who was critically ill with sepsis, pneumonia, and Cerebral Vascular Accident (CVA). Immediately after his pulmonary arterial line was pulled, he developed sudden cardiopulmonary arrest, suspected to be related to air embolism. A post-mortem CT (**a**–**c**) was performed 18 h later, revealing intravascular air in the ascending aorta (**a**, arrow), pulmonary artery (**b**, arrowhead), right ventricle (**c**, *) and the liver (arrow). Given the extent of air, it was considered most likely that the air was related to post-mortem state. Despite these findings, even when the heart was opened under water at autopsy, no air escaped, highlighting the difficulty in diagnosis of air embolism in some cases.

The cardiovascular, pulmonary and neurologic systems can suffer serious adverse effects, particularly when air enters at a high rate, which can place a strain on the right heart by raising pulmonary artery pressure. Larger air bubbles are also more likely to cause hemodynamic disturbance than smaller air bubbles [7]. With the potential for air embolism to occur almost instantaneously, it is prudent for physicians to take appropriate measures for prevention. It should also be noted that low volume air entering the venous system can often times dissipate and that air emboli can therefore spontaneously resolve on their own with minimal sequelae, (see Figure 1 and Figure 2). However, an arterial embolism may be immediately lethal. A case of air embolism is often difficult to diagnose (Figure 3) and oftentimes, the cause of an air embolism cannot be unequivocally determined. This is not unusual since cerebral air embolism is thought to be uncommon. Due to a combination of low overall incidence and some cases where the diagnosis goes undiagnosed [2], an anesthesiologist or intensive care specialist may see only a few or none during their career. A common error is for the air embolism to be misdiagnosed as a thrombotic or thrombo-embolic stroke [8]. 

## 3. Clinical Presentation Following Air Embolus

**Figure 4:**
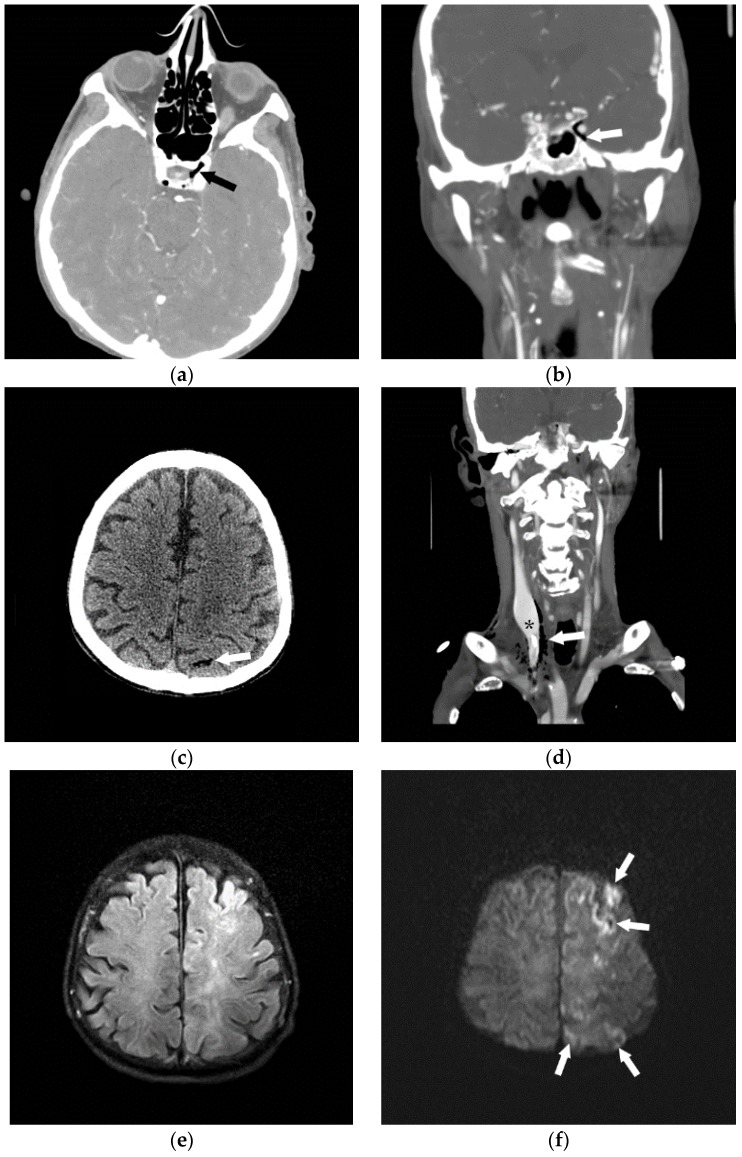

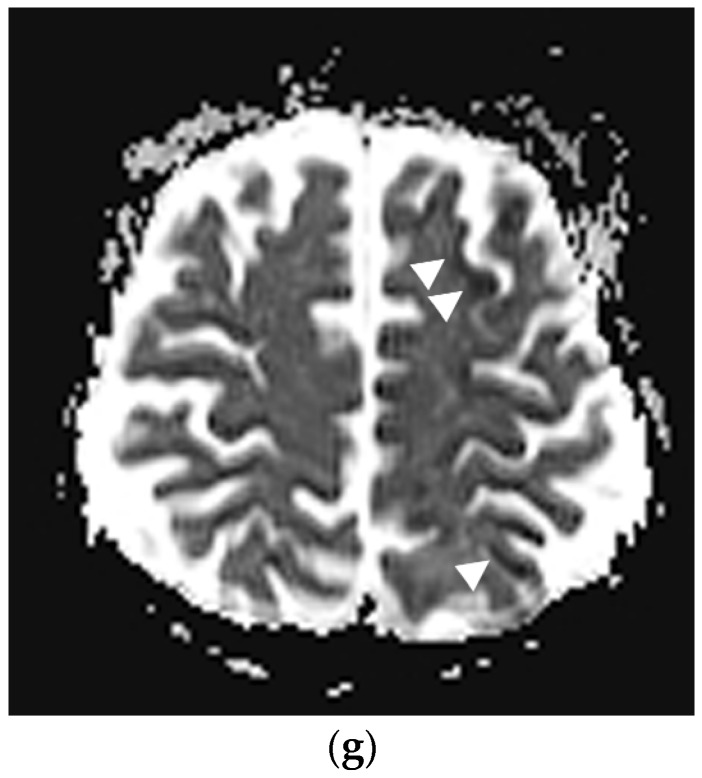
Following the placement of right internal jugular tunneled Hickman line, a 76-year-old man developed an episode of desaturation, tachycardia, and non-responsiveness. Subsequently, an immediate CT of brain, neck and chest was performed and gas was found in the left cavernous sinus (**a**,**b**) and within a left parietal sulcus (**c**); it was felt to represent air in a vessel, possibly a pial vein. There was also a large amount of air surrounding the right internal jugular vein (**d**); with a partially visualized central venous catheter (*). A brain MRI was performed showing FLAIR hyperintensity in multiple regions (**e**) with evidence of restricted diffusion (**f**); and corresponding low signal (arrowheads) on the apparent diffusion coefficient (ADC) images (**g**). The patient received a single treatment of hyperbaric oxygen but died 8 days later.

Symptoms and signs associated with serious air embolism are non-specific and can be difficult to diagnose. Clinical symptoms include dyspnea, continuous coughing and chest pain. Neurological symptoms include seizures, loss of consciousness, altered mental status, and hemiparesis/hemiplegia. In many cases, patients may exhibit sudden onset of a combination of signs and symptoms (Figure 4). Although much of the literature discusses arterial infarcts as a result of air embolism, it is worth noting that intravascular air can also cause venous infarcts [9]. 

In patients under anesthesia, reduced end-tidal CO_2_ may be noted as the earliest indicator of air embolus. Importantly, reduced oxygen saturation on pulse oximetry is considered a late sign of vascular air embolism [1]. 

**Figure 5:**
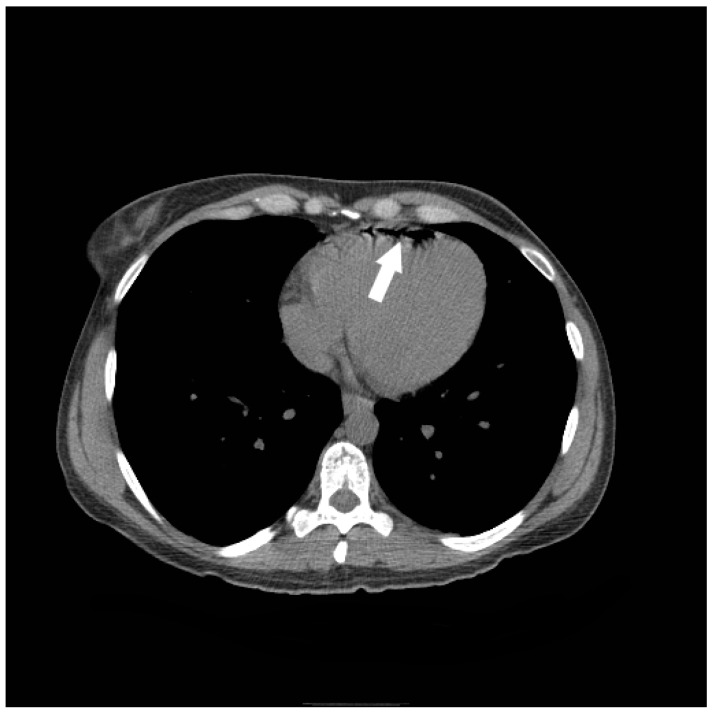
A 35-year-old woman had an incidental finding of air in the right ventricle on non-contrast CT abdomen performed for renal stone evaluation. The patient was asymptomatic and an IV line had been placed approximately 1 hour earlier.

Electrocardiogram (EKG) tracings in the presence of air embolism may show tachycardia, ST segment changes, or evidence of right heart strain. Transesophageal echocardiography can detect very small volumes of air, although its usefulness is operator-dependent [10]. Arterial blood gas analysis will show hypoxemia and hypercarbia most commonly [11]. In addition, small amounts of air may be seen relatively frequently on CT scans of the chest, where a small volume of air may enter a peripheral vein at a relatively slow rate. Such findings have been noted in up to 23% of patients, are frequently asymptomatic, and can occur with peripheral IV lines as shown in the case described in Figure 5 [12,13]. In these cases, small amounts of air travel to the pulmonary vasculature and are resorbed without sequelae.

**Figure 6:**
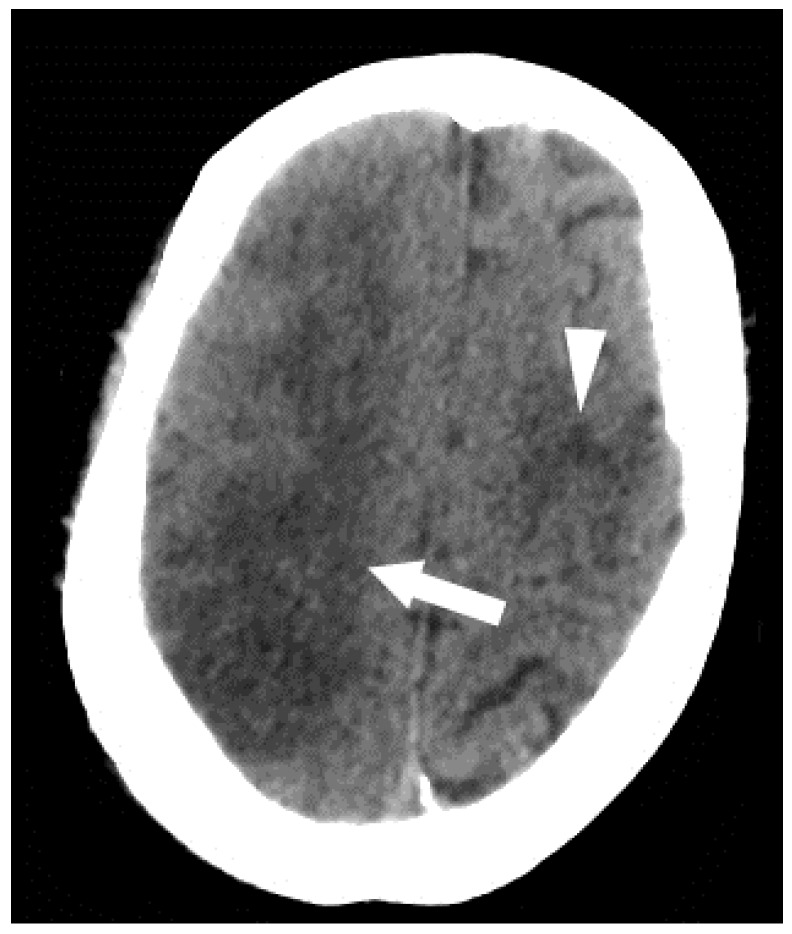
Clinical illustration of an arterial air embolism in a patient with patent foramen ovale (PFO) can be seen in the following example in which a 57-year-old female underwent liver resection. At the end of the case, the patient became profoundly hypotensive, and an air embolism was suspected. Approximately 20 cc of air was aspirated from an indwelling central venous catheter, an Extracorporeal membrane oxygenation (ECMO) was initiated and the patient was transferred to ICU where CT brain was performed. An axial CT shows multiple areas of low attenuation representing infarction of the right (arrow) and left (arrowhead) cerebral hemispheres, consistent with underlying embolic source of infarct. Furthermore, the CT shows loss of gray-white differentiation consistent with cerebral edema. Transesophageal echocardiography was performed (not shown), revealing a large amount of air bubbles in the right and left chambers heart chambers. This, together with a patent foramen ovale, resulted in paradoxical embolism.

Although small volumes of air in the venous system may be asymptomatic, this does not hold true for the arterial system, where even small amounts of air can result in end-organ damage. In those patients with a PFO, there is the potential for air to travel from the right heart to the left heart, allowing venous air to produce a paradoxical embolism, which can cause serious cerebrovascular complications (Figure 6). 

## 4. Practical Tips to Reduce the Risk of Air Embolus

### 4.1. Placing and Removing Central Venous Catheters

When placing catheters, the CVP should be raised (to decrease the pressure gradient) by placing the patient in the Trendelenburg position. It should also be ensured that patients are adequately hydrated to prevent hypovolemia and to increase CVP. Avoiding placement of venous catheters during inspiration when negative intra-thoracic pressure is at its maximum is also recommended. 

Physicians are also advised to take precautions to avoid a short subcutaneous path to the jugular vein, whenever possible. This method decreases the risk of air embolism during catheter removal [14]. When there is no guide wire in place, the operator should occlude the needle hub with their thumb. All catheter lumens should be flushed before placement and catheter hubs should be placed on tightly. It is also recommended that physicians have a working knowledge of valve mechanisms and reliability when using a peel-away sheath, use Luer–lock connections for IV ports and self-sealing valves, and that these skills are maintained by clinical staff.

When removing catheters, it is also recommended to raise CVP by keeping the patient in a supine position or with their head down or Trendelenburg position. Ideally, the venotomy site should be below the level of the heart to ensure adequate central venous pressure at the time of removal. Patients should be instructed to perform a Valsalva maneuver during catheter removal, if possible. If this is not possible, removing the catheter during active expiration is recommended [14,15]. It should be ensured that the exit site is covered with impermeable dressing and that pressure is applied afterward for 5–10 min, for hemostasis and prevention of bubble entry. It is recommended that the patient remains supine for 30 min after central venous access removal [15].

### 4.2. During an Angiogram or Other Invasive Procedure

**Figure 7:**
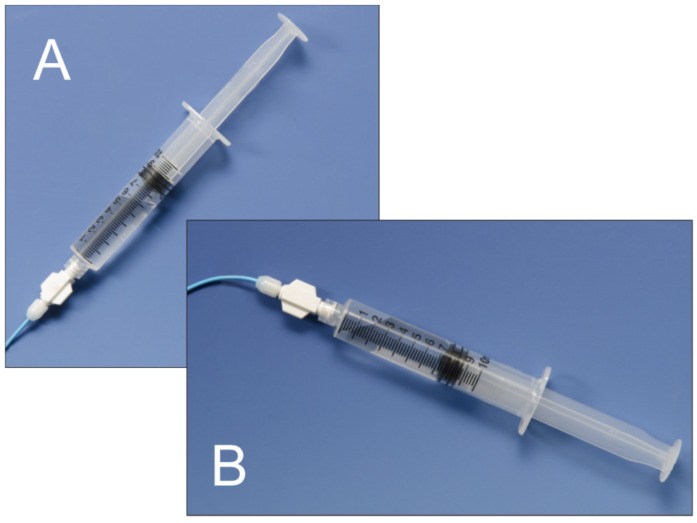
By ensuring the syringe is held with the plunger upright (**A**), there is a significant decrease in the risk of inadvertent injection of any air that may be contained within the syringe (**B**).

When conducting invasive procedures including arteriography, it is important to identify high-risk cases in advance. Use of a continuous flush through a closed system is encouraged. Any wire should be withdrawn from the catheter gradually, while using a syringe to maintain a water seal and incorporating the use of a double flush. In addition, physicians should prepare syringes in batches. This avoids having to draw and then inject and thus allows micro-bubbles to settle. All tubing should be primed with saline and no air should be present in syringes used for hand injections. The syringe should be held upright in order to ensure that any air will travel towards the syringe plunger, away from the catheter (Figure 7). To further ensure that no air is introduced into the catheter, it is recommended that the syringe be connected from wet to wet end. Additionally, it is recommended that the complete volume of the syringe never be completely injected. It should be ensured that balloons for angioplasty are correctly primed and free of air. Air-in-line detection devices incorporated into modern infusion pumps are particularly useful to detect air bubbles in plastic tubing [16]. In surgical procedures where there is a high risk of air embolism, precordial Doppler may be used during anesthesia to allow for early detection of air embolism.

Percutaneous computed tomography-guided lung biopsy is another radiologic procedure that is associated with a risk of air embolism. This is a rare complication, estimated to occur in less than one in every thousand procedures [17]. Small amounts of air that enter the pulmonary venous system have the potential to embolize to the coronary arteries, resulting in ischemia and/or cardiac arrest. Air may enter the circulation by a number of methods, including direct entry into a pulmonary vein during needle positioning and removal of the stylet. Air may also enter by direct puncture of a pulmonary arterial branch, or by puncture of an air-filled structure adjacent to a vessel, creating a fistula. In some cases, the exact cause of air embolism may be difficult to ascertain, particularly when patients are undergoing invasive procedures, as it may not always be apparent whether the air entered the operative field or from a supporting intravenous line.

## 5. Air Embolism Management

### 5.1. Initial Management Techniques 

When there is clinical suspicion of air embolism, a number of initial steps should be taken quickly to manage the situation. The initial priority is to prevent further air embolism; if air is noted entering the arterial system, the flush should be stopped immediately and the rotating hemostatic valve (RHV) should be fully opened. The arterial pressure should be allowed to passively push the air back out. This can be enhanced by turning the system vertically, which will cause air bubbles to rise. In the event of venous air embolism, the system should be dropped to minimize further entrainment of air. 

In the case of an unresponsive patient, the first priority is to address airway, breathing and circulation (ABC), including cardiopulmonary resuscitation (CPR) when necessary. Additional staff should be called to help, either in the form a “rapid response” team or as per local protocols. Once the patient has been stabilized, only then should additional evaluation and management be undertaken.

**Figure 8:**
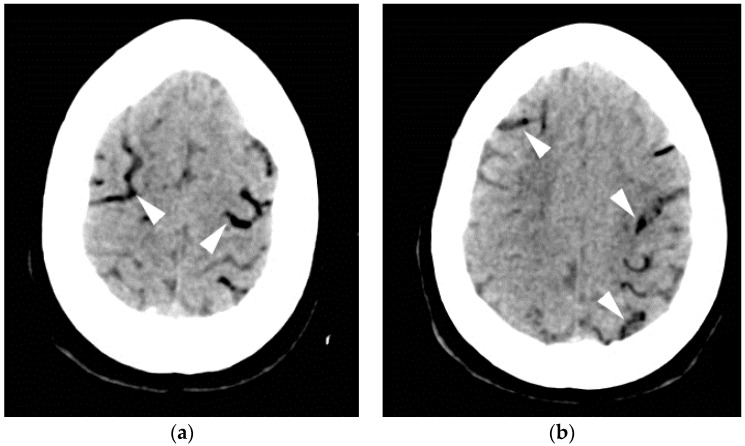
A 27-year-old woman with encephalitis who suffered a respiratory arrest. Two images from axial CT brain (**a**,**b**) performed shortly afterward revealed extensive, serpiginous hypodensity in the sulcal distribution overlying the cerebral hemispheres bilaterally (arrowheads), representing intravascular air embolization. The patient made a full recovery with prompt 100% oxygen administration and supportive measures alone.

Once stabilized, the patient should be examined (including neurological examination) and 100% oxygen treatment administered by a non-rebreather mask to maximize end-organ oxygenation. If the patient is intubated, the Fi02 should be set to 100% (Figure 8). High flow oxygen may also aid the reabsorption of nitrogen gas from the bubble into the blood, reducing the size of the air embolus [12]. 

In cases of venous air embolism, Durant’s maneuver is performed [18,19], by placing the patient in the left lateral decubitus and Trendelenburg position. This serves to encourage the air bubble to move out of the right ventricular outflow tract (RVOT) and into the right atrium, thereby relieving the “air-lock” effect responsible for potentially catastrophic cardiopulmonary collapse. It is important to note that, in the case of arterial air embolism, patients should be kept in the flat supine position as the head-down position may worsen cerebral edema [20].

**Figure 9:**
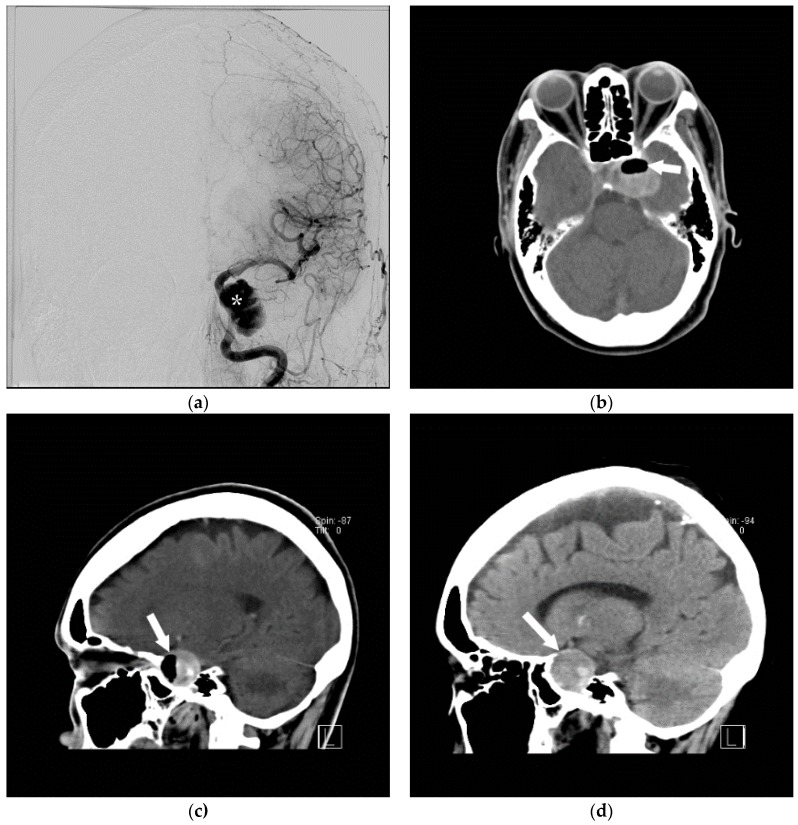
A 68-year-old woman underwent cerebral angiogram (**a**) for evaluation of 3 cm left cavernous internal carotid artery aneurysm (*). Post procedure, she developed transient aphasia, right facial droop, and right arm brachyplegia likely secondary to air embolism. This was presumed to be related to faulty occlusion balloon. The patient was immediately placed in a cervical collar to minimize head movements and distal air embolization as well as started on 100% oxygen therapy. Hyperbaric chamber therapy was discussed but declined due to concerns about patient movement during transport. CT angiogram (**b,c**) was performed immediately. Post-procedure revealed air within the large left cavernous segment internal carotid artery aneurysm (arrow). A CT performed 2 days later (**d**) showed the embolism had resolved and this patient made a full recovery.

If clinically indicated, commencement of cardiopulmonary resuscitation is warranted. This will continue end-organ perfusion and may promote migration of the air embolus into the smaller pulmonary vessels [21]. Of note, cardiopulmonary resuscitation can be challenging if the patient is positioned decubitus, or if there are femoral access catheters and sheaths for example. Patients should be transferred to an Intensive Care Unit for careful monitoring and management, and consideration should be given to hyperbaric oxygen therapy, or other advanced treatments including Extracorporeal Membrane Oxygenation (ECMO). The case in Figure 9 demonstrates the usefulness of quick and efficient initial management of air embolism. 

### 5.2. Advanced Management

Advanced hemodynamic support and monitoring requires the input of an anesthesiologist or rapid response team, who can perform advanced physiological monitoring and support such as the use of pressors and mechanical ventilation.

### 5.3. Air Aspiration

In the event of an “air lock” in the RVOT, aspiration may be required. This procedure tends to be difficult, perhaps related to the narrow luminal diameter, but offers the highest chance of success when there is already a catheter near the right atrium or ventricle. In a case report, Garg et al. outline the use of cardiac catheterization to treat an air embolism in the RVOT for a 2-year-old with Tetralogy of Fallot [22]. They use a 6 French angiographic Berman catheter to successfully aspirate the air pocket and encourage the use of catheter aspiration “in the setting of hypotension and cardiovascular collapse”. Typically, 15–20 cc of air may be aspirated using this technique. A multi side hole catheter, such as the Bunegin-Albin catheter (Cook Medical, Bloomington, IN, USA) has also been described [1,23,24]. 

## 6. Hyperbaric Oxygen Therapy

**Figure 10:**
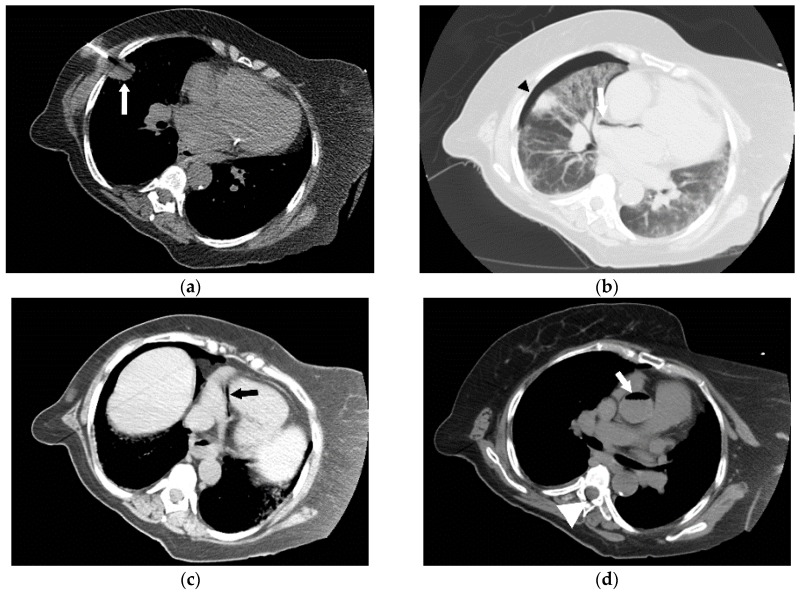

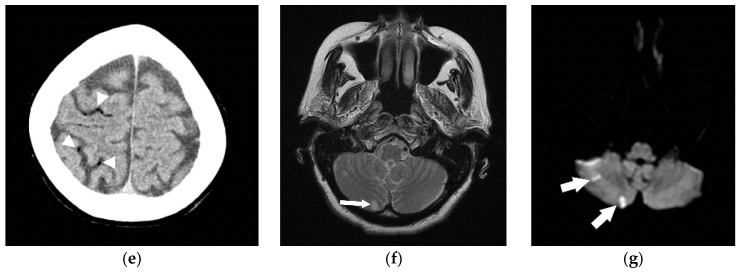
The successful use of a hyperbaric oxygen therapy (HBOT) is outlined in this case, in which a 69-year-old female with a history of suspected lung cancer underwent a CT-guided lung biopsy at an outside facility (**a**). During the procedure, the patient suffered a cardiorespiratory arrest. An immediate CT (**b**) revealed a right pneumothorax (arrow), together with air in the right pulmonary vein (arrowhead). Additional images from the CT scan (**c**,**d**), revealed air in the right coronary artery (black arrow), the ascending thoracic aorta (white arrow) and the epidural veins (arrowhead). The patient was initially unresponsive and required cardiopulmonary resuscitation. A non-contrast CT brain (**e**) was performed, revealing air scattered in the vessels overlying the right cerebral hemisphere (arrowheads). Subsequent MRI of the brain confirmed multiple areas of acute infarction in the right cerebral and cerebellar hemispheres. Axial T2 (**f**) and DWI (**g**) images of the brain demonstrated acute areas of infarction in the right cerebellum. The patient required ICU management and HBOT.

Hyperbaric oxygen therapy (HBOT) is the administration of 100% oxygen to patients at a pressure greater than sea level and is indicated in the presence of neurologic deficits. HBOT constricts pathologic air bubbles, provides oxygen to ischemic organs, and abets the conversion of nitrogen from gas to liquid phase, thereby reducing air bubble size; in essence, it diminishes gas volume, cerebral edema and enhances partial pressure of dissolved oxygen in the blood. It is widely regarded as the gold standard for treatment [25,26]. A study by Blanc et al. found that when HBOT was administered within 6 h, patients had the best outcomes [27], however treatment within 30 h may still be advantageous [28]. The successful use of HBOT is outlined in Figure 10.

## 7. Conclusions

Air embolism is rare but can be fatal and can occur during a number of invasive procedures, thus it is important that physicians are prepared to deal with this complication. Systematic planning, prompt recognition, and focused treatment offer the best chance of survival. Even with appropriate treatment, recent figures suggest that the overall one-year mortality may be in the region of 21% [29]. Radiologists and other physicians alike, should maintain a high degree of suspicion for air emboli and consider advanced management, including hyperbaric oxygen therapy, at an early stage.
